# The chain mediating role of social support and yielding coping style between health literacy and symptom burden in patients with chronic heart failure

**DOI:** 10.3389/fcvm.2025.1518175

**Published:** 2025-03-04

**Authors:** Jing Yan, Long Zhou, Guangyu Song, Yangyang Yuan

**Affiliations:** ^1^ Department of Cardiology, The First People’s Hospital of Lianyungang, Lianyungang, China; ^2^ Department of Psychiatry, The Fourth People’s Hospital of Lianyungang, Lianyungang, China

**Keywords:** chronic heart failure, health literacy, social support, coping styles, symptom burden

## Abstract

**Objective:**

To explore the mediating effects of social support and coping style on health literacy and symptom burden in patients with chronic heart failure.

**Methods:**

A total of 200 patients with chronic heart failure in Grade 3A Hospitals in Jiangsu province of China were investigated by using General Data Questionnaire, Memorial Symptom Assessment Scale for Heart Failure, Chinese Version of Heart Failure Specific Health Literacy Scale, Perceived Social Support Scale, and Medical Coping Modes Questionnaire.

**Results:**

Symptom burden score of patients with chronic heart failure was (0.73 ± 0.45), health literacy score was (29.38 ± 9.76), social support score was (59.15 ± 10.58), and yielding coping style score was (9.18 ± 4.13). There were significant correlations among health literacy, social support, coping style and symptom burden in patients with chronic heart failure. The results of mediating effect analysis showed that health literacy of patients with chronic heart failure could influence symptom burden through the chain mediating effect of social support—yielding coping style. The effect size was −0.008, and the 95% confidence interval did not include 0 (−0.011, −0.006).

**Conclusion:**

The health literacy of patients with chronic heart failure has an indirect impact on the burden of symptoms through social support and yielding coping style, suggesting that medical staff should take social support and coping style as a breakthrough from the perspective of health literacy of patients with chronic heart failure, so as to achieve the purpose of improving the health literacy of patients with chronic heart failure and reducing the burden of symptoms.

## 1 Introduction

Chronic heart failure (CHF) refers to a persistent state of heart failure, which may subsequently stabilize, worsen, or lead to decompensation. It is currently incurable and represents one of the two major challenges in the cardiovascular field of the 21st century (1). CHF is characterized by a high rate of readmission, a prolonged course, progressive worsening, and poor prognosis. Various clinical symptoms, such as fatigue, dyspnea, shortness of breath, and fluid retention, significantly inconvenience patients (2). The prevalence of heart failure in China has been rising annually, with an estimated 8.9 million cases in 2020 (3) and an estimated 12.05 million cases in 2022 (4). A report from the American Heart Association in 2023 indicates that from 2012 to 2030, the prevalence of heart failure is expected to increase by 46%, reaching epidemic proportions, with over 8 million individuals aged 18 and older affected (2). Although some studies suggest that mortality rates among CHF patients are improving, the overall mortality remains high, with a five-year mortality rate of approximately 52.6% (5). CHF imposes a significant burden on families and society and has emerged as a major global public health issue in aging populations (6).

The primary reasons for CHF patients seeking medical care or being readmitted are the onset or worsening of heart failure symptoms. The physiological signs associated with symptom onset or exacerbation can provide early warning of impending decompensation, allowing for timely and effective identification of adverse prognostic information (7). Due to blood stasis, CHF leads to widespread changes in the cardiovascular system throughout the body, and patients may experience a variety of symptoms over a period of time. These symptoms collectively contribute to the symptom burden. ‘Symptom burden’ is defined as ‘the overall subjective experience of heart failure symptoms that negatively impact the patient or their family, including symptom prevalence, frequency, and severity’ (8). A study involving 1,353 CHF patients found that the most prevalent symptoms were chest pain (81.67%), shortness of breath (71.69%), and fatigue (68.51%) (9). The symptoms in CHF patients are interrelated and interact, causing significant physiological and psychological distress (2, 9). In CHF patients, symptom burden is associated with various health outcomes (10). As treatment goals increasingly focus on symptom management, improving quality of life, and extending lifespan, there is considerable research on the relationship between symptom burden and quality of life in CHF patients. However, research on the factors influencing symptom burden and exploration of related mechanisms remains insufficient.

Limited health literacy is widespread, to the extent that health literacy assessment needs to be considered in clinical practice settings. Health literacy has emerged as a new area of research in chronic disease management and is considered the sixth vital sign (11). The ‘Healthy China Action (2019–2030)’ (12) released by the Central Committee of the Communist Party of China and the State Council emphasizes that improving health literacy levels is one of the most fundamental, cost-effective, and efficient measures to enhance overall health. The document sets a broad objective of significantly raising national health literacy levels and markedly reducing premature mortality rates caused by major chronic diseases by 2030. As a comprehensive evaluation indicator reflecting the development of the national health sector, health literacy has been incorporated into the national health development plan, indicating that integrating health literacy strategies into chronic disease clinical research aligns with the evolving needs of the times. Existing research has shown that health literacy can influence the onset and progression of disease symptoms, with higher levels of health literacy acting as a protective factor against the emergence of adverse symptoms (13). Health literacy affects health outcomes through various mechanisms (14). However, there is currently a relative paucity of studies, both domestically and internationally, examining the impact of health literacy on symptom burden in patients with CHF. Clarifying the interplay between health literacy and symptom burden in CHF patients, as well as understanding the intermediary pathways through which health literacy affects symptom burden, is essential for targeted interventions aimed at enhancing health literacy and improving symptom burden in this population.

Social support is an available external resource that is closely related to an individual's physiological and psychological health, playing a crucial role in symptom management for patients with CHF. Relevant studies have indicated that social support can negatively predict symptoms in CHF patients, including both physiological and psychological symptoms (15). Health literacy has a significant predictive effect on social support (16), and it directly influences the level of social support received (17). Scholars abroad have suggested that future research should consider the impact of health literacy on the effectiveness of social support (18). However, it is necessary to explore whether social support interacts with health literacy to influence symptom burden in CHF patients. Additionally, further investigation is warranted to determine whether social support acts as a mediator between health literacy and symptom burden.

Coping styles are important mediating variables in the psychological stress process, reflecting the attitudes and strategies individuals adopt in response to stress and pressure. They play a critical role in patients’ psychological regulation processes (19). Research has shown that health literacy indirectly influences patients’ disease-related behaviors by directly affecting their coping styles, including problem-focused, avoidance, and yielding coping styles (20). Positive coping styles can enhance disease management knowledge and skills, alleviate adverse emotions, and reduce disease burden (21). The level of health literacy can predict coping styles, with negative coping styles showing a significant correlation with disease-related distress (22). Additionally, coping tendencies have been found to be significantly negatively correlated with patients’ symptom burden (23). Therefore, whether coping styles mediate the relationship between health literacy and symptom burden in CHF patients requires further investigation.

This study hypothesizes that health literacy influences symptom burden in CHF patients through social support and coping styles. To test this hypothesis, a mediating effect model will be employed for analysis. The aim is to provide a foundational basis for the targeted promotion of health literacy among CHF patients and to improve their symptom burden.

## 2 Materials and methods

### 2.1 Subjects

Using a convenience sampling method, this study selected patients with CHF who were hospitalized in the cardiovascular department of a tertiary hospital from March 2023 to December 2023 as the research subjects. Inclusion Criteria: (1) Patients diagnosed with CHF by attending physicians or higher, based on the diagnostic criteria outlined in the ‘2018 Chinese Guidelines for the Diagnosis and Treatment of Heart Failure’ (24). Patients classified as New York Heart Association (NYHA) functional class II to IV according to the American College of Cardiology's classification requirements. (2) Patients aged 18 years or older. (3) Patients who provided informed consent and voluntarily agreed to participate in this study.Exclusion Criteria: (1) Patients with severe wasting diseases or malignant tumors. (2) Patients with mental disorders or communication difficulties. (3) Patients who have undergone heart transplantation. Sample Size Estimation: Based on Kendall's sample size calculation method, the number of samples selected should be 5–10 times the number of study variables (25), with a minimum sample size of 135 cases. Considering potential invalid or missing questionnaires during the data collection process, a 20% increase in the sample size was applied, resulting in a final collection of 200 valid questionnaires, based on the annual hospitalization rate of CHF patients at the hospital.

### 2.2 Questionnaire

#### 2.2.1 General data questionnaire

The main variables include gender, age, marital status, educational level, average monthly income, method of medical payment, body mass index (BMI), heart function classification, self-assessment of salt intake, and physical activity level.

#### 2.2.2 Memorial symptom assessment scale-heart failure (MSAS-HF)

The scale was developed by American nursing scholar Zambroski (26). and consists of a total of 32 items, with each item representing a specific symptom across three dimensions: physiological symptoms, psychological symptoms, and heart failure symptoms. Among these, 26 items are assessed in terms of frequency (1–4 points), severity (1–4 points), and distress (0–4 points). The remaining 6 items are evaluated only based on severity (1–4 points) and distress (0–4 points).To ensure consistency in the scoring of the three characteristics of each symptom item, the distress levels of ‘0’, ‘1’, ‘2’, ‘3’ and ‘4’ are assigned values of ‘0.8’, ‘1.6’, ‘2.4’, ‘3.2’ and ‘4’ respectively. The mean of the three characteristics represents the score for each symptom item. If a patient does not experience a particular symptom item, the score for that item is recorded as ‘0’. The total score for the patient's symptoms is calculated as the average of all the item scores on the scale, with higher scores indicating a greater symptom burden for the patient. In this study, the overall Cronbach's *α* coefficient for the scale was 0.861.

#### 2.2.3 Chinese version of heart failure specific health literacy scale (C-HFS-HLS)

The scale was developed by Japanese scholar Matsuoka (27) and consists of 12 items, divided into three dimensions: functional health literacy, interactive health literacy, and critical health literacy. Each item is scored on a scale from 1 to 4, where 1 indicates ‘not applicable,’ 2 indicates ‘rarely applicable,’ 3 indicates ’sometimes applicable,’ and 4 indicates ‘very applicable.’ Items 1 to 4 are reverse scored, while the remaining items are scored in a positive direction. The total score for the scale is 48 points, with higher scores indicating a higher level of health literacy. In this study, the overall Cronbach's *α* coefficient for the scale was 0.956.

#### 2.2.4 Perceived social support scale (PSSS)

The scale was developed by Zimet (28) and consists of 12 items categorized into three dimensions: family support, friend support, and other support. Each item uses a 1–7 Likert scale, where scores range from 1 (‘strongly disagree’) to 7 (‘strongly agree’). The total score for the 12 items is 84 points, with higher scores indicating a higher level of perceived social support. The score ranges from 12 to 36, 37 to 60, and 61 to 84, which correspond to low, moderate, and high levels of social support, respectively. In this study, the overall Cronbach's α coefficient for the scale was 0.970.

#### 2.2.5 Medical coping modes questionnaire (MCMQ)

The questionnaire was developed by foreign scholars Feifel (29). It has a total of 20 items, including three dimensions: facing, avoiding, and yielding. Each item is scored from 1 to 4, of which items 1, 4, 9, 10, 12, 13, 18, and 19 are negatively scored, and the remaining items are positively scored. Higher total scores in each dimension indicate that patients are more likely to adopt that particular coping mode. In this study, the Cronbach's α coefficients for the facing dimension, avoidance dimension, and yielding dimension were 0.917, 0.923, and 0.962, respectively.

### 2.3 Data collection

The questionnaires were distributed on-site, accompanied by standardized instructions guiding patients to complete them independently. Upon completion, the questionnaires were collected for verification to ensure their completeness and validity. A total of 210 questionnaires were distributed, with 200 valid responses collected, resulting in an effective response rate of 95.2%.

### 2.4 Statistical analysis

SPSS 26.0 software was used for statistical analysis. The study variables (health literacy, symptom burden, social support, and coping styles) were continuous numerical variables that conformed to a normal distribution. Descriptive analysis was presented using the mean and standard deviation; otherwise, the median and interquartile range were reported. Correlation analysis was conducted using Pearson correlation. Mediation analysis was performed using the Process plugin in SPSS, employing bootstrap methods to test the significance of the mediation effect. If the confidence interval of the test results did not include 0, this indicated a significant mediation effect; conversely, if the interval included 0, the mediation effect was considered not significant.

## 3 Results

### 3.1 The general information of participants

Among 200 patients with CHF, there were 113 male patients (56.5%) and 87 female patients (43.5%). Patients aged 70 years and older numbered 97 (48.5%), those aged 46–69 years totaled 91 (45.5%), and 12 patients (6.0%) were aged 18–45. A total of 163 patients were married (81.5%), and 118 patients (59%) had an education level of elementary school or junior high school or lower, while 39 patients (19.5%) had a college degree or higher. Eighty-two patients (41%) reported an average monthly income of over 5,000 yuan. The living situation indicated that 146 patients (73%) resided in urban areas, and 108 patients (54%) cohabited with children, spouses, or parents. There were 135 patients (67.5%) covered by health insurance. In terms of heart function classification, 113 patients (56.5%) were classified as New York Heart Association (NYHA) Class II, 66 patients (33%) as Class III, and 21 patients (10.5%) as Class IV. 105 patients (52.5%) had a Body Mass Index (BMI) of 24 kg/m^2^ or greater, and 153 patients (76.5%) had preserved ejection fraction. A total of 111 patients (55.5%) had one to two comorbidities, and 79 patients (39.5%) had undergone health examination every one to two years. Additionally, 94 patients (47%) self-reported normal or average daily salt intake, while 124 patients (62%) did not engage in regular exercise (Table 1).

**Table 1:** Demographic and clinical characteristics of patients with CHF (*n* = 200).

Item	Frequency	Proportion (%)
Sex		
Male	113	56.5
Female	87	43.5
Age		
18–45	12	6.0
46–69	91	45.5
≥70	97	48.5
Marital status		
Unmarried	6	3.0
Married	163	81.5
Divorced/separated/widowed	31	15.5
Education		
No school	31	15.5
Primary school/Junior high school	87	43.5
High school/Technical secondary school	43	21.5
College degree and above	39	19.5
Average monthly income		
≤1,000 yuan	40	20.0
1,001–4,999 yuan	78	39.0
≥5,000 yuan	82	41.0
Type of permanent residence		
Rural	54	27.0
City	146	73.0
Living situation		
Living alone	9	4.5
Living with a spouse	83	41.5
Living with a spouse as well as parents or children	108	54.0
Medical payment method		
Self-funded/public funded	8	4.0
Health Insurance	135	67.5
Cooperative Medical Care	57	28.5
BMI (kg m^2^)		
<18.5	10	5.0
18.5–23.9	85	42.5
24–27.9	70	35.0
≥28	35	17.5
Heart function		
II	113	56.5
III	66	33.0
IV	21	10.5
Ejection fraction		
Reduce	47	23.5
Reserve	153	76.5
Types of comorbidity		
0	36	18.0
1–2	111	55.5
≥3	53	26.5
Frequency of health examination		
Average 1–2 years	79	39.5
Average 3–5 years	64	32.0
No regular check-ups	57	28.5
Self-assessment of salt intake		
More (>6 g)	84	42.0
Normal/General (3–6 g)	94	47.0
Less (<3 g)	22	11.0
Regular exercise		
Yes	76	38.0
No	124	62.0

### 3.2 Current status of symptom burden, health literacy, social support, and coping styles in CHF patients

In this study, the symptom burden score of CHF patients was 0.73 ± 0.45, the health literacy score was 29.38 ± 9.76, the social support score was 59.15 ± 10.58, the facing coping style score was 19.45 ± 5.53, the avoidance coping style score was 12.78 ± 4.62, and the yielding coping style score was 9.18 ± 4.13 (Table 2).

**Table 2:** Symptom burden, health literacy, social support, and coping style scores of CHF patients (*n* = 200).

Variable	Minimum	Maximum	Mean	Standard deviation
MSAS -HF				
Physical symptoms	0.00	1.63	0.56	0.38
Psychological symptoms	0.00	2.39	0.68	0.62
Symptoms of heart failure	0.00	3.31	0.96	0.58
Symptom burden score	0.00	2.15	0.73	0.45
C-HFS-HLS				
Functional health literacy	4.00	16.00	10.48	4.02
Interactive health literacy	4.00	16.00	10.99	2.95
Critical health literacy	4.00	16.00	7.92	3.77
Health Literacy	13.00	48.00	29.38	9.76
PSSS				
Family support	8.00	28.00	23.43	3.70
Friends support	8.00	28.00	17.74	4.05
Additional support	8.00	28.00	17.98	3.83
Social support	36.00	80.00	59.15	10.58
MCMQ				
Face	8.00	32.00	19.45	5.53
Avoid	7.00	25.00	12.78	4.62
Yield	5.00	20.00	9.18	4.13

MSAS-HF, memorial symptom assessment scale-heart failure; C-HFS-HLS, Chinese version of heart failure specific health literacy scale; PSSS, perceived social support scale; MCMQ, medical coping modes questionnaire.

### 3.3 Correlation analysis among health literacy, social support, coping style, and symptom burden in CHF patients

The results of pearson correlation analysis showed that the symptom burden scores of CHF patients in this study were closely related to health literacy, social support, and coping style scores. Among them, the health literacy of patients was significantly negatively correlated with the symptom burden score (*r* = −0.557, *P* < 0.01), social support was significantly negatively correlated with the symptom burden score (*r* = −0.680, *P* < 0.01), and the yielding coping style was significantly positively correlated with the symptom burden score (*r* = 0.774, *P* < 0.01). In addition, the health literacy of CHF patients was significantly positively correlated with the social support score (*r* = 0.597, *P* < 0.01), and was significantly negatively correlated with the yielding coping style score (*r* = −0.646, *P* < 0.01), at the same time, the social support of CHF patients was significantly negatively correlated with the yielding coping style score (*r* = −0.718, *P* < 0.01) (Table 3).

**Table 3:** Correlation analysis of health literacy, social support, coping style, and symptom burden in CHF patients.

Variable	Health literacy	Social support	Yielding coping style	Symptom burden
Health literacy	1			
Social support	0.597^a^	1		
Yielding coping style	−0.646^a^	−0.718^a^	1	
Symptom burden	−0.557^a^	−0.680^a^	0.774^a^	1

^a^ Indicates significant correlation at the 0.01 level (two-tailed).

### 3.4 Test of the chain mediating effect of social support and yielding coping style between health literacy and symptom burden in CHF patients

**Figure 1:**
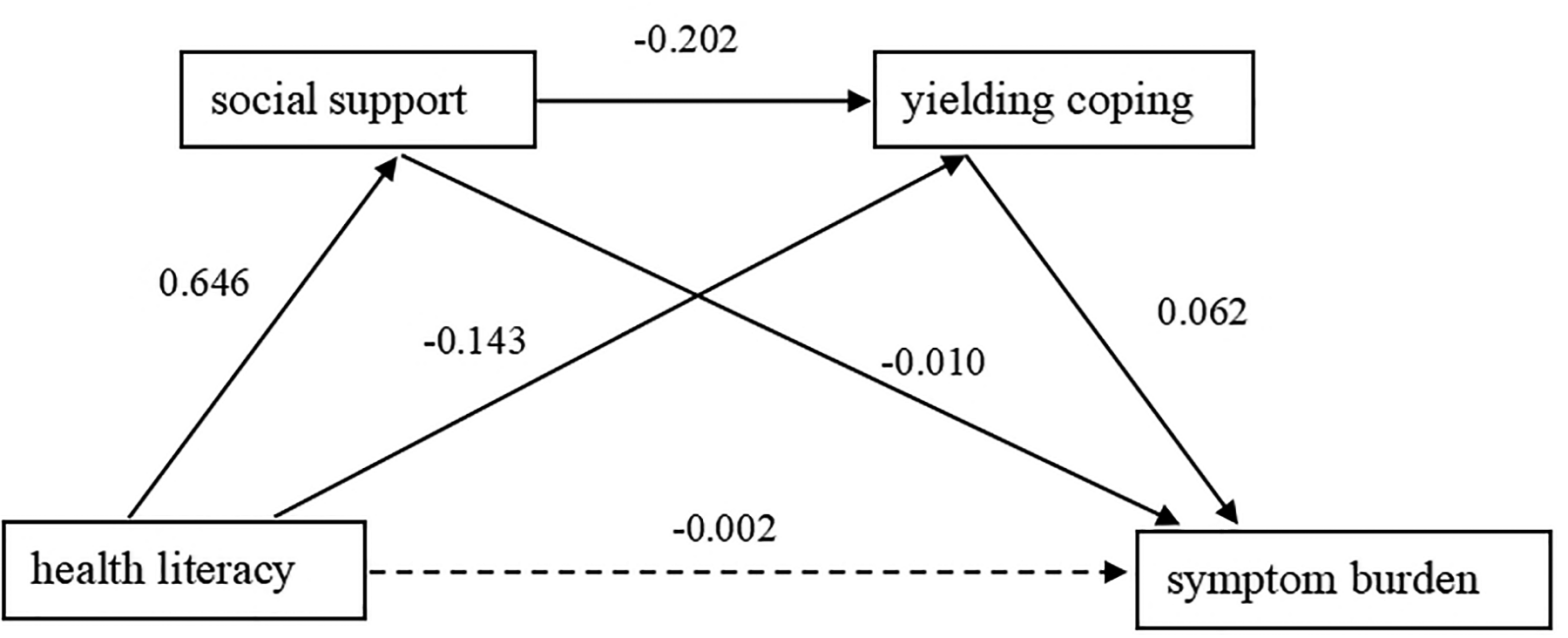
Chain mediation model of social support and yielding coping between health literacy and symptom burden in CHF patients.

With the health literacy of CHF patients as the independent variable, symptom burden as the dependent variable, social support and yielding coping style as the mediating variables, a chain mediation effect model was constructed (Figure 1), and the model 6 in the process plug-in was used for analysis and testing (Table 4).

**Table 4:** Test of the mediating effect of social support and yielding coping between health literacy and symptom burden in CHF patients.

Regression equation		*R* ^2^	F	Coeff	t	95% CI	
Outcome variable	Predictor variables					LLCI	ULCI
Social support	Health literacy	0.356	109.423***	0.646	10.461***	0.524	0.768
Yielding coping	Social support	0.590	141.460***	−0.202	−9.091***	−0.246	−0.158
	Health literacy			−0.143	−5.933***	−0.190	−0.095
Symptom burden	Social support	0.632	111.953***	−0.010	−3.790***	−0.016	−0.005
	Yielding coping			0.062	8.433***	0.048	0.077
	Health literacy			−0.002	−0.735	−0.007	0.003

Coeff is the regression coefficient, *p < 0.05, **p < 0.01, ***p < 0.001.

Health literacy of CHF patients can significantly positively predict social support, with a coefficient of 0.646 (95% CI: 0.524, 0.768), and significantly negatively predict the yielding coping style, with a coefficient of −0.143 (95% CI: −0.190, −0.095), but has no significant effect on symptom burden, with a coefficient of −0.002, *P* > 0.05, 95% CI is (−0.007, 0.003), including 0. Social support can significantly negatively predict the yielding coping style, with a coefficient of −0.202 (95% CI: −0.246, −0.158), and significantly negatively predict the symptom burden, with a coefficient of −0.010 (95% CI: −0.016, −0.005), the yielding coping style can significantly positively affect the symptom burden of CHF patients, with an impact coefficient of 0.062 (95% CI: 0.048, 0.077).

The mediating effect of CHF patients’ health literacy on symptom burden through social support is −0.007. The Bootstrap test shows that the 95% CI is (−0.011, −0.003), which does not include 0, proving that the mediating effect is significant; the effect of CHF patients health literacy on symptom burden through yielding coping style is −0.009. The Bootstrap test shows that the 95% CI is (−0.013, −0.006), which does not include 0, proving that the mediating effect is significant, the chain mediating effect of CHF patients’ health literacy on symptom burden through social support and yielding coping style is −0.008. The Bootstrap test shows that the 95% CI is (−0.011, −0.006), which does not include 0, proving that the chain mediating effect is significant (Table 5).

**Table 5:** Analysis of the mediating effects of social support and yielding coping between health literacy and symptom burden in CHF patients.

Path relationship	Effect size	95% CI	
		LLCI	ULCI
Total indirect effect	−0.024	−0.029	−0.019
CHF health literacy-social support-symptom burden	−0.007	−0.011	−0.003
CHF health literacy-yielding coping-symptom burden	−0.009	−0.013	−0.006
CHF health literacy-social support-yielding coping-symptom burden	−0.008	−0.011	−0.006

## 4 Discussion

Through this study, the symptom burden characteristics and health literacy levels of CHF patients can be better understood. The hypothesis that health literacy influences symptom burden through social support and coping styles is generally supported.

### 4.1 Health literacy and symptom burden levels of CHF patients

In this study, among the three dimensions of symptom burden in patients with CHF, the heart failure symptom dimension scored the highest, consistent with the findings of domestic scholar Xu Xiaohua (30). This indicates that a majority of CHF patients experience typical heart failure symptoms, primarily related to cardiac dysfunction. The psychological symptom dimension received the next highest score, which may be attributed to the generally low educational level of CHF patients, lack of proper health guidance, and the chronic nature of their illness. Research by international scholars, including Strangl (31) also found that CHF patients commonly experience various negative psychological symptoms, which impact their quality of life. In contrast, the physiological symptom dimension score in this study was lower than that reported by Xu Xiaohua, primarily because 56.5% of the 200 study participants had a heart function classification of II, with a majority having preserved ejection fraction. In Xu Xiaohua's study, however, a greater proportion of participants had heart function classifications of III (64.04%) and IV (10.86%), with over half (51.69%) exhibiting reduced ejection fraction. This further suggests that the symptom burden in CHF patients may be significantly correlated with their functional classification and ejection fraction levels.

In this study, the health literacy score of patients with CHF was 29.38 ± 9.76, which is similar to the findings of domestic researchers Zheng Zhanzhan (32), but lower than those reported by Japanese scholars Matsuoka (27). This discrepancy may be attributed to the fact that 59% of the participants in this study had an education level of junior high school or below, indicating generally low educational attainment, and a higher proportion of elderly individuals aged over 70. Due to declines in physiological function and cognitive abilities, older adults often have a poor understanding of their health conditions. Coupled with their limited educational background, this leads to inadequate comprehension of health information related to heart failure, thereby affecting the overall health literacy level of these patients. Furthermore, the results of this study indicate that the interactive health literacy dimension scored relatively high among CHF patients, followed by functional health literacy, with the critical health literacy dimension scoring the lowest. This finding is consistent with several domestic studies (33, 34). It suggests that CHF patients are more inclined to acquire health information through interaction and communication with others. This emphasizes the importance for healthcare providers to enhance verbal communication and educational efforts with patients in clinical settings, aiding them in accessing more health information. In contrast to the first two dimensions, critical health literacy imposes higher demands on patients. Therefore, it is crucial for healthcare professionals to ensure that health education for CHF patients is not merely about imparting information. Instead, it should focus on guiding and encouraging patients to apply critical thinking skills in analyzing health information, enabling them to make informed health decisions that are appropriate to their individual circumstances.

### 4.2 Analysis of mediation effect

#### 4.2.1 Analysis of the mediating effect of social support between health literacy and symptom burden in CHF patients

Health literacy represents a personal ability, which is a protective factor against some adverse symptoms of the disease (35), and social support is equivalent to a kind of external force, which positively contributes to symptom management behaviors, and both health literacy and social support interact with each other to alleviate the symptomatic burden that CHF brings to patients. In terms of the content of social support, CHF patients with high health literacy often take the initiative to communicate with family members, friends, and others both materially and emotionally. At the time of presentational analysis, this study found that CHF patients had relatively strong interactive health literacy skills among the three dimensions, although their health literacy level was not high. Therefore, it is suggested that healthcare professionals can improve the overall health literacy level of CHF patients by increasing communication with them, and they should also pay attention to the material and emotional needs of patients and give full play to the mediating role of social support. For example, patients should be guided to learn to talk about their troubles and actively seek help from others when difficulties occur; health guidance should be provided to patients’ families, and family members should be taught and encouraged to participate in assisting in symptom management (36), and a patient support group should be established to initiate peer communication and support among patients. Improving the health literacy of CHF patients and seeking more social support, on both tracks, will have better results in improving the symptom burden.

#### 4.2.2 Analysis of the mediating effect of yielding coping style between health literacy and symptom burden in CHF patients

The symptom burden experienced by patients with CHF includes psychological dimensions, where physiological and psychological symptoms interact and collectively distress the patient. Research indicates that nearly half of patients experience negative psychological issues, such as depression (37). From the perspective of passive coping strategies, patients with lower health literacy often feel powerless against their illness, believing that recovery is unattainable. This resignation leads them to succumb to their condition, refraining from taking proactive measures and adopting a fatalistic attitude, which further exacerbates their symptom burden. Thus, it is essential for healthcare providers to prioritize health literacy education for CHF patients in clinical practice, while also focusing on assessing and intervening in their coping strategies. For CHF patients who tend toward passive coping styles, it is important to provide guidance and support related to their condition. Increased communication can help them adjust their mindset and develop a correct and positive understanding of their illness. Moreover, addressing the needs of these patients is crucial for enhancing their treatment adherence, allowing for timely management of adverse symptoms. By empowering them to confront the physiological and psychological changes associated with their disease, patients can learn to face challenges rather than succumbing to the threat of their illness. Ultimately, this approach aims to alleviate the symptom burden of the disease and improve their quality of life (22).

#### 4.2.3 Analysis of the chain mediating effect of social support and yielding coping style between health literacy and symptom burden in CHF patients

This study found that social support and yielding coping play multiple mediating roles between health literacy and symptom burden in patients with CHF. The chain mediation effect was significant, further elucidating the mechanism by which health literacy impacts symptom burden in CHF patients. Specifically, the findings indicate that lower health literacy in CHF patients positively predicts lower social support, which in turn negatively predicts the use of yielding coping strategies. Ultimately, these yielding coping strategies have a positive effect on the symptom burden, suggesting that patients with lower health literacy experience lower levels of social support and, consequently, are more likely to adopt yielding coping strategies, leading to a heavier symptom burden. Patients with higher health literacy demonstrate greater subjective initiative, actively seeking external resources and utilizing them effectively (38), thereby achieving higher levels of social support. Moreover, support from family, friends, and other significant individuals is a key factor affecting patients’ coping mechanisms. Improving the coping strategies of patients suffering from severe physical and psychological symptoms requires not only a positive attitude towards their condition but also the collaborative efforts of family members, friends, and healthcare professionals to establish health-related support and resources (39).

However, after the four variables were included in the model, the direct effect of health literacy on symptom burden was not significant, but the mediating effects were significant. That is, in this study, health literacy only acted on symptom burden through the mediating effects of social support and yielding coping style. It can be seen that social support and yielding coping style have stronger direct effects on symptom burden. Therefore, taking the improvement of patients’ social support and coping style as a breakthrough point has a more direct and significant effect on targeted improvement of health literacy and reduction of disease symptom burden. In clinical work, in order to achieve the goal of reducing the symptom burden of CHF patients, we can start from the perspective of health literacy. While educating health literacy, doctors, nurses, patients, families and other parties should cooperate (40) to improve their social support and encourage them to adopt positive coping methods to face the disease, thereby reducing the symptom burden.

## 5 Conclusion

The symptom burden in patients with CHF is significantly correlated with health literacy, social support, and coping styles. Social support and yielding coping style play a chain mediating role between health literacy and symptom burden. This indicates that health literacy in CHF patients has an indirect impact on symptom burden through social support and yielding coping. These findings suggest that healthcare providers should focus on improving health literacy from the perspective of CHF patients, using social support and coping style as key intervention points. Emphasizing the improvement of health literacy can enhance social support and coping strategies, ultimately aiming to effectively increase health literacy and reduce symptom burden in patients with CHF.

## Data Availability

The original contributions presented in the study are included in the article/Supplementary Material, further inquiries can be directed to the corresponding author.

## References

[1] Skrzypek A Mostowik M Szeliga M Wilczyńska-Golonka M Dębicka-Dąbrowska D Nessler J. Chronic heart failure in the elderly: still a current medical problem. Folia Med Cracov. (2018) 58(4):47–56. 30745601

[2] McDonagh TA Metra M Adamo M Arango JE Cowie MR Böhm M 2021 ESC guidelines for the diagnosis and treatment of acute and chronic heart failure: developed by the task force for the diagnosis and treatment of acute and chronic heart failure of the European Society of Cardiology (ESC). With the special contribution of the heart failure association (HFA) of the ESC. Eur J Heart Fail. (2021) 24(1):4–131. 10.1002/ejhf.2333 35083827

[3] The Writing Committee of the Report on Cardiovascular Health and Diseases in China. Overview of the report on cardiovascular health and diseases in China 2021. Chin J Circ. (2022) 37(06):553–78. 10.3969/j.issn.1000-3614.2022.06.001 39401992

[4] Wang H Li Y. Comprehensive management of patients with worsening chronic heart failure: Chinese expert consensus 2022. Chin J Circ. (2022) 37(03):215–25. 10.3969/j.issn.1000-3614.2022.03.003

[5] Heidenreich PA Bozkurt B Aguilar D Allen LA Byun JJ Colvin MM 2022 AHA/ACC/HFSA guideline for the management of heart failure: a report of the American College of Cardiology/American Heart Association joint committee on clinical practice guidelines. Circulation. (2022) 145(18):e895–e1032. 10.1161/CIR.0000000000001063 35363499

[6] Wang H Chai K Du M Wang S Cai JP Li Y Prevalence and incidence of heart failure among urban patients in China: a national population-based analysis. Circ Heart Fail. (2021) 14(10):e008406. 10.1161/CIRCHEARTFAILURE.121.008406 34455858

[7] Maqsood MH Khan MS Warraich HJ. Association of palliative care intervention with health care use, symptom burden and advance care planning in adults with heart failure and other noncancer chronic illness. J Pain Symptom Manage. (2021) 62(4):828–35. 10.1016/j.jpainsymman.2021.02.017 33631325

[8] Stockdill ML Patrician PA Bakitas M. Understanding and measuring symptom burden in heart failure: a concept analysis. West J Nurs Res. (2019) 41(10):1423–47. 10.1177/0193945919833710 30895895

[9] Liu Y Hao R Chen H Tian H Yang Q. A multicenter cross-sectional study on the relationship between symptom clusters and quality of life in patients with chronic heart failure. Chin J Chronic Dis Prev Control. (2022) 30(7):507–511+516. 10.16386/j.cjpccd.issn.1004-6194.2022.07.006

[10] Santos GC Liljeroos M Dwyer AA Jaques C Girard J Strömberg A Symptom perception in heart failure—interventions and outcomes: a scoping review. Int J Nurs Stud. (2021) 116:103524. 10.1016/j.ijnurstu.2020.103524 32063295

[11] Rodts ME Unaka NI Statile CJ Madsen NL. Health literacy and caregiver understanding in the CHD population. Cardiol Young. (2020) 30(10):1439–44. 10.1017/S1047951120002243 32746956

[12] Han D Gao HX Hou GL. Policy Analysis of the ‘Healthy China Action (2019–2030)’ from the perspective of policy instruments. Med Soc. (2020) 33(11):20–4. 10.13723/j.yxysh.2020.11.004

[13] Liu XW Qin WZ Xu LZ Zhang J Gao ZR Hu FF The relationship between electronic health literacy and depressive symptoms among residents in tai'an city. Chin J Mental Health. (2022) 36(5):427–32. 10.3969/j.issn.1000-6729.2022.05.012

[14] Kim H Xie B. Health literacy in the eHealth era: a systematic review of the literature. Patient Educ Couns. (2017) 100(6):1073–82. 10.1016/j.pec.2017.01.015 28174067

[15] Wang F. The impact of symptom burden on quality of life in patients with chronic heart failure and model construction (Master’s thesis). Bengbu Medical College, China (2016).

[16] Ma D Ren J Qiao G Cheng W Yun Y Ma Y. Correlation between health literacy, social support, and health beliefs in patients with bladder cancer undergoing urinary diversion and stoma creation. J Nurs. (2021) 36(17):33–5. 10.3870/j.issn.1001-4152.2021.17.033

[17] Yu Q Lei L Wan B Fu L Zhao X Zeng Q Correlation between health literacy and core knowledge of tuberculosis prevention and control, and social support in tuberculosis patients. Chin J Antituberculosis. (2020) 42(11):1227–31. 10.3969/j.issn.1000-6621.2020.11.015

[18] Jo A Ji SE Son YJ. The roles of health literacy and social support in improving adherence to self-care behaviours among older adults with heart failure. Nurs Open. (2020) 7(6):2039–46. 10.1002/nop2.599 33072389 PMC7544858

[19] Su YD Mo YH Liu L Wang XH. Type D personality and psychological stress in patients with chronic heart failure: the mediating roles of coping styles and psychological resilience. Chin J Nurs. (2022) 39(3):19–23. 10.3969/j.issn.1008-9993.2022.03.005

[20] Liu YF Zhu LM Liu HW Wu YQ. Analysis of health literacy status and influencing factors among caregivers of liver cancer patients. J Interv Radiol. (2024) 33(2):191–6. 10.3969/j.issn.1008-794X.2024.02.017

[21] Gong Y Wang P Zheng XM. The mediating role of interdependence in the relationship between family resilience, positive emotions, and active coping strategies in patients with chronic heart failure. J Pract Heart Lung Vasc Dis. (2021) 29(12):47–52. 10.12114/j.issn.1008-5971.2021.00.277

[22] Li SC Chen HT Zhang HM Fu R. The chain mediating effect of social support and coping strategies on self-efficacy and diabetes distress in young patients with type 2 diabetes. J Nurs Manag. (2022) 22(10):718–22. 10.3969/j.issn.1671-315x.2022.10.005

[23] Pang JM. The relationship between type D personality and symptom burden in patients with chronic heart failure: the mediating effects of social support and coping tendencies (Master’s thesis). Shandong University, China (2019).

[24] Chinese Society of Cardiology Heart Failure Group, Chinese Medical Doctor Association Heart Failure Committee and Editorial Board of Chinese Journal of Cardiovascular Diseases. Chinese Guidelines for the diagnosis and treatment of heart failure 2018. Chin J Cardiovasc Dis. (2018) 46(10):760–89. 10.3760/cma.j.issn.0253-3758.2018.10.004

[25] Bujang MA. An elaboration on sample size determination for correlations based on effect sizes and confidence interval width: a guide for researchers. Restor Dent Endod. (2024) 49(2):21. 10.5395/rde.2024.49.e21 PMC11148401 38841381

[26] Zambroski CH Moser DK Bhat G Ziegler C. Impact of symptom prevalence and symptom burden on quality of life in patients with heart failure. Eur J Cardiovasc Nurs. (2005) 4(3):198–206. 10.1016/j.ejcnurse.2005.03.010 15916924

[27] Matsuoka S Kato N Kayane T Yamada M Koizumi M Ikegame T Development and validation of a heart failure-specific health literacy scale. J Cardiovasc Nurs. (2016) 31(2):131–9. 10.1097/JCN.0000000000000226 26049813

[28] Zimet GD Powell SS Farley GK Werkman S Berkoff KA. Psychometric characteristics of the multidimensional scale of perceived social support. J Pers Assess. (1990) 55(3-4):610–7. 10.1080/00223891.1990.9674095 2280326

[29] Feifel H Strack S Nagy VT. Coping strategies and associated features of medically ill patients. Psychosom Med. (1987) 49(6):616–25. 10.1097/00006842-198711000-00007 3423168

[30] Xu X Liu R Lin Y. The path of electronic health literacy on symptom burden in patients with chronic heart failure. Chin J Military Med. (2020) 37(12):14–7. 10.3969/j.issn.1008-9993.2020.12.004

[31] Strangl F Ischanow E Ullrich A Oechsle K Fluschnik N Magnussen C Symptom burden, psychosocial distress and palliative care needs in heart failure: a cross-sectional explorative pilot study. Clin Res Cardiol. (2023) 112(1):49–58. 10.1007/s00392-022-02017-y 35420358 PMC9849173

[32] Zheng Z Yang B Song T Li X. Analysis of health literacy status and influencing factors in patients with chronic heart failure. Chin Nurs Management. (2019) 19(1):23–9. 10.3969/j.issn.1672-1756.2019.01.008

[33] Sang M. A study on the impact of health literacy education on self-management in patients with chronic heart failure (Master’s thesis). Tianjin University of Traditional Chinese Medicine, China (2020).

[34] Wen B. Research on the current status of health literacy levels and influencing factors in patients with chronic heart failure (Master’s thesis). Dalian Medical University, China (2022).

[35] Eronen J Paakkari L Portegijs E Saajanaho M Rantanen T. Health literacy supports active aging. Prev Med. (2021) 143:106330. 10.1016/j.ypmed.2020.106330 33220399

[36] Babygeetha A Devineni D. Social support and adherence to self-care behavior among patients with coronary heart disease and heart failure: a systematic review. Eur J Psychol. (2024) 20(1):63–77. 10.5964/ejop.12131 38487598 PMC10936663

[37] Kinugasa Y Adachi T Fukuki M Hirota Y Ishiga N Kato M Factors affecting the willingness of nursing care staff to cooperate with heart failure care and the role of internet video education. J Gen Fam Med. (2023) 25(1):19–27. 10.1002/jgf2.658 38239992 PMC10792320

[38] Ng A Wong F. Effects of a home-based palliative heart failure program on quality of life, symptom burden, satisfaction, and caregiver burden: a randomized controlled trial. J Pain Symptom Manage. (2018) 55(1):1–11. 10.1016/j.jpainsymman.2017.07.047 28801001

[39] Son YJ Lee HJ. Association between persistent smoking after a diagnosis of heart failure and adverse health outcomes: a systematic review and meta-analysis. Tob Induc Dis. (2020) 18:5. 10.18332/tid/116411 PMC6986333 31997987

[40] Gheiasi SF Cheraghi MA Dastjerdi M Navid H Khoshavi M Peyrovi H Experiences of facilitators and inhibitors to treatment adherence in patients with heart failure. Clin Nurs Res. (2023) 32(3):648–59. 10.1177/10547738221147402 36788432

